# Colon Cancer Metastasis to the Right Testis: Case Report and Review of Literature

**DOI:** 10.1155/2022/2649259

**Published:** 2022-08-18

**Authors:** Mizuki Kasahara, Tomo Shimizu, Hiroshi Aoki, Mizuho Okawa, Fumito Yamabe, Hideyuki Kobayashi, Koichi Nagao, Koichi Nakajima, Yozo Mitsui

**Affiliations:** Department of Urology, Toho University Faculty of Medicine, 143-8540 Tokyo, Japan

## Abstract

**Introduction:**

A metastatic testicular tumor is uncommon. We report here a case of testicular metastasis associated with recurrent colorectal cancer. *Case Presentation*. A 75-year-old male was presented with right scrotum pain one year after undergoing a right hemicolectomy combined with resection of the small intestine and omentum for ascending colon cancer (pT4N0M0). Magnetic resonance imaging of the pelvis showed a 7.3 × 5.4 × 4.5 cm mass consisting of a cystic solid tumor. A right inguinal orchiectomy was performed and right testicular pain improved after surgery. Pathology results showed that the tumor was a metastatic adenocarcinoma. The patient subsequently died two months later due to progression of the colon cancer.

**Conclusion:**

Although colorectal cancer metastasis to the testis is very uncommon, it should be kept in mind in clinical situations, especially for older males with a testicular mass or discomfort.

## 1. Introduction

A metastatic carcinoma to the testis is rare, accounting for only 0.8-2.3% of all testicular tumors [1, 2], with the most common primary site of the prostate gland, accounting for approximately 40% of all reported cases [3]. Here, details are presented regarding the treatment of a patient with a painful right testicular tumor found to be a metastasis from ascending colon cancer surgically treated one year prior. A right inguinal orchiectomy was performed for providing a pathological diagnosis and reducing pain.

## 2. Case Presentation

**Figure 1:**
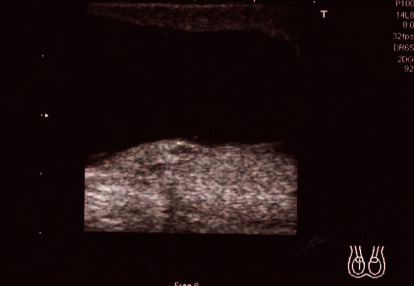
Scrotal ultrasonography image showing liquid component. Neoplastic lesions, normal testicle components, and the epididymis were not clearly visible.

**Figure 2:**
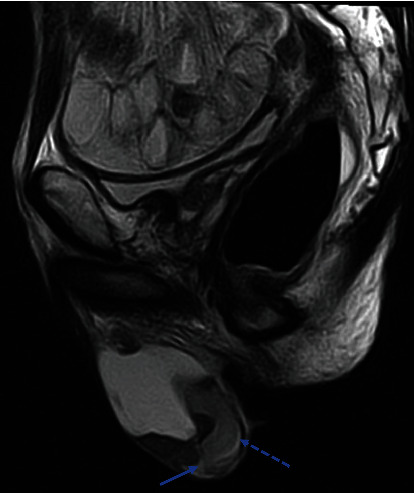
MRI T2-weighted image of the pelvis showing a 7.3 × 5.4 × 4.5 cm testicular mass consisting of a cystic solid tumor (arrow) as well as normal testicle components (dashed line arrow).

A 75-year-old male was referred to our hospital for right scrotal pain that was initially noted three weeks prior. One year before referral, the patient had undergone a right hemicolectomy combined with resection of the small intestine and omentum for ascending colon cancer (pT4N0M0) and subsequently received adjuvant chemotherapy with oxaliplatin for three cycles. Following that treatment, he did not visit the treating hospital regularly for follow-up examinations. Our physical examination findings revealed right scrotal swelling as well as tenderness. Scrotal ultrasonography showed a liquid component in the scrotum, while neoplastic lesions, normal testicle components, or the epididymis were not clearly visualized (Figure 1). A scrotal puncture was performed followed by a physical examination, during which palpation revealed a mass above the testis to the epididymis along with remaining scrotal tenderness. Magnetic resonance imaging (MRI) of the pelvis was then performed for reevaluation, which showed a 7.3 × 5.4 × 4.5 cm cystic solid tumor in the right scrotum, suspected to be a seminoma or malignant lymphoma (Figure 2, T2-weighted image). Laboratory results revealed normal or slightly elevated levels of prostate-specific antigen, alpha-fetoprotein, carbohydrate antigen 19-9, soluble inteleukin-2 receptor, beta-human gonadotrophin, and lactate dehydrogenase, while those of carcinoembryonic antigen (1,174 ng/mL) and cancer antigen 125 (56 U/mL) were markedly elevated. In addition, computed tomography (CT) findings indicated the presence of multiple metastatic tumors in the lungs, liver, lymph nodes, skin, and abdominal cavity. Based on the imaging and laboratory results, it was considered that colorectal cancer had recurred and metastasized throughout the body.

**Figure 3:**
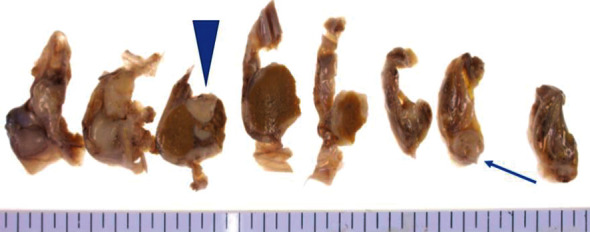
Macroscopic views of the resected specimen showing ill-defined white solid mass found above the testis (arrow) and in the epididymis (arrowhead).

**Figure 4:**
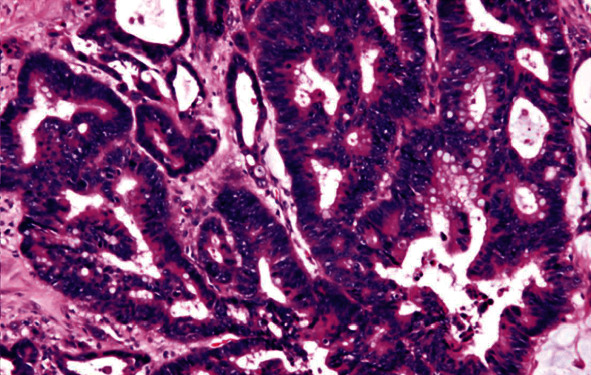
Histological findings of resected testis showed an adenocarcinoma with moderate differentiation, identical to the primary colon cancer (H&E stain, ×200).

The patient was subsequently admitted to our hospital for pain control and anorexia. Right scrotal pain did not improve after administering a narcotic preparation for systemic pain, thus a right orchiectomy via an inguinal approach was performed for pain relief and diagnosis, during which a part of the scrotal wall was resected to treat adhesions. As shown in Figure 3, an ill-defined white solid mass was found above the right testis (3.0 cm) and another in the epididymis (1.3 cm). Histopathological results also showed an adenocarcinoma with moderate differentiation, which was identical to the primary colon cancer (Figure 4). In addition, cancer cells were found disseminated on the serosal surface of the peritoneal sheath of the spermatic cord and extended to the dartos fascia at the bottom of the scrotum. Based on these findings, the mass in the right testis was diagnosed as a metastatic tumor from primary colon cancer. Right scrotal pain improved after surgery, though the patient died two months later due to cancer progression.

## 3. Discussion

Metastasis of a carcinoma to the testis is rare and accounts for only 0.8-2.3% of all testicular tumors. The diagnosis is most often incidental in an autopsy examination [1, 2, 4]. Occurrence has been noted in males from 29 to 90 years old; the prostate is the most common primary site, followed by the lung, melanoma, and the kidney [4]. A previous study estimated that metastatic testicular tumors from digestive organs comprised 17.5% of all such tumors, among which colorectal cancer is the most likely to metastasize to a testicular cancer, though fewer than 80 cases have been reported thus far [5, 6].

Most patients with a metastatic testicular tumor from colon cancer are presented with testicular swelling or a hydrocele, with or without pain, and the mass is not solid [1, 7]. Thus, careful diagnostic testing is necessary, as this disease can be confused with a benign or inflammatory condition. Diagnosis of metastasis of carcinoma to the testis is generally confirmed based on microscopy results, though other important factors including patient age, history of present or prior malignancy, and results obtained with imaging modalities such as ultrasonography, CT, or MRI can assist with the final determination. In the present case, important information regarding the scrotum was easily obtained with minimally invasive ultrasonography, while MRI results provided evidence of the testicular tumor in greater details. CT imaging is also useful for assessing the spread of cancer cells to systemic organs, while increased gastrointestinal tumor markers in the present case was an important finding strongly suggesting colon cancer recurrence/metastasis. Thus, a multidisciplinary approach based on patient history, blood sampling data, and use of various imaging modalities can contribute to the diagnosis of a metastatic testicular tumor.

Although details regarding the mechanism of testicular metastasis of colon cancer remain largely unknown, several candidate theories have been suggested [7, 8], including arterial embolization, retrograde venous or lymphatic spread, and direct spread. Interestingly, it was recently proposed that microscopic channels of communication may be present between the peritoneum and testes, because most cases are presented with a testicular hydrocele [4], indicating that cancer cells may invade and extend from the original or a disseminated lesion through microscopic sized channels between the peritoneum and testes. The present findings also support this hypothesis from the perspective of hydrocele presence, though additional investigations are needed to clarify the precise mechanisms of metastatic carcinoma development in the testis.

Resection performed for liver or lung metastasis from primary colon cancer can improve prognosis in some cases [9, 10]. However, the significance of surgical treatment for testicular metastasis from colon cancer is unclear, since that is regarded as a late manifestation of a systemic spread of cancer cells. Indeed, in most cases with an initial presentation of colon cancer as testicular metastasis, an average survival was found to be 6-12 months regardless of treatment [1]. However, if the site is symptomatic, as in the present case, we think that a surgical approach should be considered for improving patient's quality of life.

In summary, we experienced a rare case of right testicular metastasis from colon cancer, for which the patient was treated with inguinal orchiectomy. Although metastasis to the testis is rare, awareness of the possibility is important for clinicians, especially when an older male is presented with a testicular mass or discomfort. Moreover, surgical treatment should be considered for relief of symptoms.

## Additional Points

*Keynote Message.* We encountered a rare case of right metastatic testicular tumor from the ascending colon cancer in a patient who was presented with pain in that area. Local treatment for testicular metastasis does not typically prolong survival, because the condition is generally a late manifestation of systemic spreading of cancer cells. However, surgical removal of a metastatic testis is reasonable for improving quality of life such as relief of pain as well as providing findings for a definitive diagnosis.
